# Variation across analysts in statistical significance, yet consistently small effect sizes

**DOI:** 10.1073/pnas.2218957120

**Published:** 2023-01-09

**Authors:** Maya B. Mathur, Christian Covington, Tyler J. VanderWeele

**Affiliations:** ^a^Quantitative Sciences Unit and Department of Pediatrics, Stanford University, Palo Alto, CA 94304; ^b^Department of Biostatistics, Harvard University, Boston, MA 02115; ^c^Department of Epidemiology, Harvard University, Boston, MA 02115

Breznau et al. assessed variation in results when 73 research teams analyzed the same dataset to investigate whether greater immigration reduces public support for social policies (1). Breznau et al. reported “massive variation in reported results,” including “widely diverging numerical findings.” These conclusions were based on variation in statistical significance across estimates (e.g., 25% of estimates were significant and negative, 58% were nonsignificant, and 17% were positive and significant). Research teams also reported different subjective conclusions, which closely aligned with whether their estimates were significant.

However, considering variation in effect sizes rather than only variation in statistical significance suggests different conclusions. The point estimates themselves varied minimally across teams, and nearly all estimates were quite close to the null. For example, 90% of standardized estimates were within the range [−0.037, 0.037]; that is, 90% of estimates suggested that a one-unit increase in immigration was associated with an increase or decrease in public support of <4% of a SD. While standardized effect sizes can be difficult to interpret and are inherently contextual (2), these effect sizes are more than fivefold smaller than classic benchmarks defining “small” effect sizes (3) and do not seem to support unqualified conclusions of “massive variation.” We would encourage Breznau et al. to grapple with whether, in substantive context, effect sizes falling within this narrow range around the null truly exhibit massive variation.

A well-known problem with exclusive focus on statistical significance is that, for very large datasets, even a very small point estimate can be significant. Breznau et al.’s teams analyzed a dataset containing tens of thousands of observations, which explains the “substantial variation” in estimates’ significance even though the estimates themselves were consistently quite small and close to the null. Given the minimal variation in the estimates, it is also not entirely surprising that little of this variation could be systematically explained by identifiable analytic decisions. If there is little heterogeneity in the true population effect sizes underlying the point estimates, then much of the remaining variation in point estimates may simply be statistical error that would be accurately captured in the estimates’ individual confidence intervals (CIs). Variation in estimates due to statistical error is not part of a “hidden universe” of decisions but rather is fully captured by appropriate statistical inference, including CIs. One can also use meta-analytic methods to estimate variation in population effect sizes, excluding statistical error (4, 5). This analysis suggests that 90% of standardized population effects would be in an even narrower range around the null of [−0.014, 0.014] (i.e., effects of < 2% of a SD).

The replication crisis has helped revive long-standing, compelling injunctions to stop degrading evidence into categories of “significant” versus “not significant” (6). Researchers should assess evidence using measures such as effect sizes, CIs, and *P* values treated as continuous values (or Bayesian analogs). Unfortunately, meta-researchers assessing variation in results across analysts or replication studies have themselves sometimes focused entirely on variation in significance; as we have seen here, this practice can lead to potentially misleading conclusions.

## Reproducibility

All data and R code required to reproduce these results are publicly available (https://github.com/ctcovington/MCV_BRWN-22_comment).
